# Improving lung cancer risk stratification leveraging whole transcriptome RNA sequencing and machine learning across multiple cohorts

**DOI:** 10.1186/s12920-020-00782-1

**Published:** 2020-10-22

**Authors:** Yoonha Choi, Jianghan Qu, Shuyang Wu, Yangyang Hao, Jiarui Zhang, Jianchang Ning, Xinwu Yang, Lori Lofaro, Daniel G. Pankratz, Joshua Babiarz, P. Sean Walsh, Ehab Billatos, Marc E. Lenburg, Giulia C. Kennedy, Jon McAuliffe, Jing Huang

**Affiliations:** 1 Veracyte, Inc., South San Francisco, CA 94080 USA; 2 Section of Computational Biomedicine, Department of Medicine, Boston University School of Medicine, Boston, MA 02118 USA; 3 Department of Statistics, University of California, Berkeley, Berkeley, CA 94720 USA

**Keywords:** Lung cancer, Bronchoscopy, Risk stratification, Gene expression, Whole transcriptome RNA sequencing, Machine learning, Molecular diagnostic test

## Abstract

**Background:**

Bronchoscopy for suspected lung cancer has low diagnostic sensitivity, rendering many inconclusive results. The Bronchial Genomic Classifier (BGC) was developed to help with patient management by identifying those with low risk of lung cancer when bronchoscopy is inconclusive. The BGC was trained and validated on patients in the Airway Epithelial Gene Expression in the Diagnosis of Lung Cancer (AEGIS) trials. A modern patient cohort, the BGC Registry, showed differences in key clinical factors from the AEGIS cohorts, with less smoking history, smaller nodules and older age. Additionally, we discovered interfering factors (inhaled medication and sample collection timing) that impacted gene expressions and potentially disguised genomic cancer signals.

**Methods:**

In this study, we leveraged multiple cohorts and next generation sequencing technology to develop a robust Genomic Sequencing Classifier (GSC). To address demographic composition shift and interfering factors, we synergized three algorithmic strategies: 1) ensemble of clinical dominant and genomic dominant models; 2) development of hierarchical regression models where the main effects from clinical variables were regressed out prior to the genomic impact being fitted in the model; and 3) targeted placement of genomic and clinical interaction terms to stabilize the effect of interfering factors. The final GSC model uses 1232 genes and four clinical covariates – age, pack-years, inhaled medication use, and specimen collection timing.

**Results:**

In the validation set (*N* = 412), the GSC down-classified low and intermediate pre-test risk subjects to very low and low post-test risk with a specificity of 45% (95% CI 37–53%) and a sensitivity of 91% (95%CI 81–97%), resulting in a negative predictive value of 95% (95% CI 89–98%). Twelve percent of intermediate pre-test risk subjects were up-classified to high post-test risk with a positive predictive value of 65% (95%CI 44–82%), and 27% of high pre-test risk subjects were up-classified to very high post-test risk with a positive predictive value of 91% (95% CI 78–97%).

**Conclusions:**

The GSC overcame the impact of interfering factors and achieved consistent performance across multiple cohorts. It demonstrated diagnostic accuracy in both down- and up-classification of cancer risk, providing physicians actionable information for many patients with inconclusive bronchoscopy.

## Background

The National Lung Screening Trial showed that low dose computed tomographic (CT) screening results in a mortality reduction through early detection of lung nodules [1], but suffers a high rate of false positive findings, resulting in additional diagnostic procedures to reach a definitive diagnosis. Compared to other procedures, bronchoscopy is a relatively safe and less invasive diagnostic tool for the evaluation of lung nodules, with low complication rates [2]. However, the reported sensitivity of bronchoscopy is as low as 34% and varies widely depending on the location and size of the lesion, which results in a suboptimal diagnostic yield [3]. Patients with nondiagnostic bronchoscopy results are often referred for additional invasive procedures, including surgical lung biopsy and transthoracic needle biopsy. To reduce the rate and risk of these invasive procedures, complementary and less invasive approaches are needed to improve the overall diagnostic yield after bronchoscopy and to provide physicians with more actionable information.

Using a microarray-based gene expression platform, the Bronchial Genomic Classifier (BGC) was originally developed to assess the risk of lung cancer in current and former smokers with a nondiagnostic bronchoscopy [4, 5]. The BGC leverages the molecular “field of injury” that occurs in airway epithelial cells exposed to cigarette smoke [6]. Gene expression changes in the airway epithelium were found to correlate with the presence of cancerous lung nodules in current and former smokers [7]. The BGC was designed to be a “rule-out” test, with high sensitivity to detect malignancy and a negative predictive value over 90% when patients with an intermediate pre-test cancer risk are reclassified as low (post-test) risk. Test performance was validated in two independent cohorts [4, 5]; and clinical utility of the BGC was demonstrated in the same cohorts [8] by modeling the potential reduction in invasive procedures among patients who were down-classified from intermediate pre-test to low post-test risk of lung cancer.

In this study, we developed a second-generation risk stratification algorithm for lung cancer – the Genomic Sequencing Classifier (GSC) –with broadened utility beyond the BGC. Like the BGC, the GSC continues to leverage the molecular field of injury whereby cigarette smoking creates gene expression changes in bronchial airway epithelial cells, some of which are associated with lung cancer [6, 7]. As cigarette smoking impacts the bronchial airway transcriptome differently among current vs. former smokers [6], smoking status (current vs. former) is now recognized to be a dominant factor explaining the overall bronchial airway gene expression variations. The genome-wide impact of smoking status makes it challenging to distinguish genomic lung cancer signals from smoking effect in bronchial airway epithelium. To better address such a challenge, we developed a genomic smoking index to capture the smoking effect shared among subjects with and without lung cancer, and then included it as a covariate in algorithm development.

In developing the GSC, we utilized samples from over 1600 patients from several clinical cohorts, all of whom underwent bronchoscopy after suspicious lung nodules were detected on HRCT and most of whom (96%) were current or former smokers. Due to the large number of samples, multiple sequencing batches needed to be processed over a period of time and by multiple laboratory operators. Therefore, technical batch effects among processing runs needed to be minimized. This was achieved through a two-step approach: early detection of potential batch effects using replicated control samples carefully placed throughout development phase processing runs, and removal of unwanted variation with algorithmic optimization.

Combining multiple independent cohorts posed unique challenges: the most critical one being distribution shifts in key clinical factors. In addition, we found that differences in airway epithelial gene expression were linked to the timing of bronchial brushing specimen collection during the bronchoscopy procedure. The proportion of patients with sample collection prior to, versus after, other cytology and/or pathology sampling during bronchoscopy was substantially different across cohorts and this imbalance was associated with gene expression differences across cohorts. Moreover, the sequencing data used for classifier development is from upper airway bronchial epithelial cells, which are not direct samplings of the lung lesion itself. It is therefore not surprising that a genomic signal differentiating malignant from benign would be relatively modest, and potentially masked or confounded by stronger signals such as those due to smoking damage response, or age and other clinical characteristics. To compensate for this, we leveraged interaction terms between clinical and genomic features to better manage the impact from these factors and to maintain overall classification performance across cohorts. An ensemble classification approach was utilized to stabilize performance across patients with different clinical characteristics and multiple batches in sequencing.

Here we describe the identification and mitigation of technical and analytical challenges in developing the GSC using next generation whole transcriptome RNA sequencing data. We demonstrate that the GSC achieved robust performance across different clinical cohorts for both down- and up- classification of lung cancer risk. We propose potential clinical utility for nodule management in patients with inconclusive bronchoscopy results.

## Methods

### Study design

This study utilized bronchial brushing samples from three cohorts of current and former smokers who underwent bronchoscopy for suspected lung cancer: the Airway Epithelial Gene Expression in the Diagnosis of Lung Cancer (AEGIS-1 and AEGIS-2) [4], and the prospective BGC Registry study [9]. A total of 1718 subjects with a suspicious lung nodule were enrolled in the AEGIS study and 576 subjects were enrolled in the BGC Registry study (Additional file 1 Fig. S1) at the time of algorithm development. Subjects were excluded from the development and validation of the classifiers due to missing clinical data, low RNA sample quantity, or a sequencing QC failure. Subjects within indication are defined as those satisfying these criteria: they are current or former smokers, they had nondiagnostic bronchoscopy results and they do not have a history of former or concurrent cancer. All other subjects are considered out of indication (OOI) and they include never smokers, subjects with malignancy (lung cancer) detected at bronchoscopy, subjects with cancers metastatic to the lung, and subjects with a history of former or concurrent cancer. In AEGIS, 435 subjects were within indication. The assignment of AEGIS subjects to training and test cohorts used in the BGC validation was preserved here [4, 5]. In the BGC Registry cohort, 288 subjects were within indication, and were randomly split into 122 subjects in the training set and 166 subjects in the test set (Additional file 1 Fig. S1). In the combined set of AEGIS and BGC Registry cohorts, there were 1361 subjects out of indication, including 947 subjects with malignancy determined at bronchoscopy, 63 never smokers and 203 subjects with a history of former or concurrent cancer. All out-of-indication subjects were assigned to the training set since they were informative in understanding genomic features such as cancer signals, smoking damage and epithelial cell types.

All subjects were followed until a diagnosis was established, or for 12 or 24 months after bronchoscopy. A diagnosis of lung cancer was established at the time of bronchoscopy or subsequently by additional diagnostic procedures. Benign disease was determined based on a review of medical records and follow-up procedures at 12 months post-bronchoscopy [4, 5].

### Molecular testing, sequencing pipeline, and data QC

Bronchial brushing samples were collected during bronchoscopy and were stored in a nucleic acid preservative (RNAprotect, QIAGEN, Hilden, Germany) before shipment to Veracyte for processing. From each brushing sample, total RNA was extracted using the miRNeasy Mini Kit (QIAGEN, Hilden, Germany), quantitated (QuantiFluor RNA System, Promega, Madison, WI) and 50 ng was used as input to the TruSeq RNA Access Library Prep procedure (Illumina, San Diego, CA) for coding transcriptome enrichment. Libraries meeting quality control criteria were sequenced using NextSeq 500 instruments (2 × 75 bp paired-end reads) with the High Output Kit (Illumina, San Diego, CA). Raw sequencing (FASTQ) files were aligned to the Human Reference assembly 37 (Genome Reference Consortium) using the STAR RNA-seq aligner software [10]. Uniquely mapped and non-duplicate reads were summarized for 63,677 annotated Ensembl genes using HTSeq [11]. Data quality metrics were generated using RNA-SeQC [12]. Samples were excluded and re-sequenced when their library sequence data did not achieve minimum criteria for total reads, uniquely mapped reads, mean per-base coverage, base duplication rate, percentage of bases aligned to coding regions, base mismatch rate and uniformity of coverage within each gene. To monitor potential technical batch effects, four commercially acquired cell lines and lung tissue samples (control samples) and bronchial brushing samples from five patients (sentinel samples) were included in each 96 well plate across all sequencing runs. The sentinel samples were selected from the commercial stream of the BGC with diverse clinical features and gene expression representative of the patient population. Kinship analysis [13] was performed on all samples with acceptable sequencing quality metrics to ensure sample identity.

### Genomic gender

We developed a genomic gender index to verify that samples sequenced were in agreement with the corresponding clinical annotation. The genomic gender index uses raw expression counts from 9 genes on chromosome Y and 611 genes on chromosome X, and is calculated as follows:

$$ Genomic\ gender\ index=\frac{\varSigma_i\ {\mathit{\log}}_2\left({y}_i+1\right)}{{\mathit{\log}}_2\left({\varSigma}_j{x}_j\right)}, $$

where and *x*_*j*_ ′ *s* and *y*_*i*_ ′ *s* are raw counts for genes on chromosome X and Y, respectively. The genomic gender of a sample is “Male” if the index is greater than 3.16, and “Female” otherwise. Mentions of gender hereafter in this paper refer to genomic gender.

### Normalization and gene filtering

Sequence data were filtered to exclude any features that were not targeted for enrichment by the library assay, resulting in 26,268 Ensembl genes. Expression count data at the gene level were normalized by the variance stabilizing transformation (VST) method in DESeq2 [14]. We used BGC Registry training samples that were within indication as the seed set, which is a frozen dataset used to estimate the dispersion-mean relationship of gene expression. Dispersions were fit using the “local” option and considering subject diagnosis (benign/malignant label), clinical smoking status and specimen collection timing. For each individual sample in the training set and the independent test set, the sample is combined and normalized with the seed set samples. Size factors and normalization factors, which are sample-level and gene x sample level adjustment scale, were estimated using the seed set samples as reference. Normalization factors for individual genes were estimated separately for the plus strands and the minus.

From the 26,268 gene expression features profiled by the assay platform [15, 16], we identified 7991 genes with less than 1 transcript per million in over 99% of all samples in the training set, which were hereafter defined as low expressers and excluded from feature selection. Meanwhile, we identified 369 genes with excessive variability under constant assay conditions, thus potentially affected by batch effects, using replicated control and sentinel samples processed under similar technical conditions (i.e. batches) using the RUV method [17]. After removal of the low expressers and batch-sensitive genes, a total of 17,954 genes were included in subsequent analyses.

### Gene expression correlation analysis

We applied Weighted Gene Co-expression Network Analysis (WGCNA) [18, 19] on the gene expression profiles of bronchial brushing samples from within-indication subjects (*N* = 311), to examine the gene expression patterns and their association with subject diagnosis (lung cancer or benign disease), clinical factors (current or former smoker, pack-years, current use of inhaled medication), specimen collection timing, and cohort (AEGIS or BGC Registry). Genes on sex chromosomes were removed from this analysis. Normalized (VST) gene expression data were used to construct a co-expression network and module eigengenes (the first principal component of individual gene modules). We examined over-represented biological processes in each gene module using PANTHER [20].

### Genomic smoking index

Several previous studies showed reversible and irreversible effects of smoking on epithelial gene expression, with distinguishable patterns between current versus former smokers [6, 21]. A binary classification model for current or former smoker status was trained using 1438 samples with clinical smoking status from the training portion of AEGIS and BGC Registry cohorts, augmented with 140 samples from an additional independent study cohort [22]. The input features were normalized gene expression levels after exclusion of genes with low expression or affected by batch effects. Five-fold cross-validation (CV) with five repeats were used to evaluate training performance of the genomic smoking index. Sample partition for cross-validation was balanced by smoking status (current vs. former), pre-test risk (low, intermediate and high) and cohort.

**Fig. 1:**
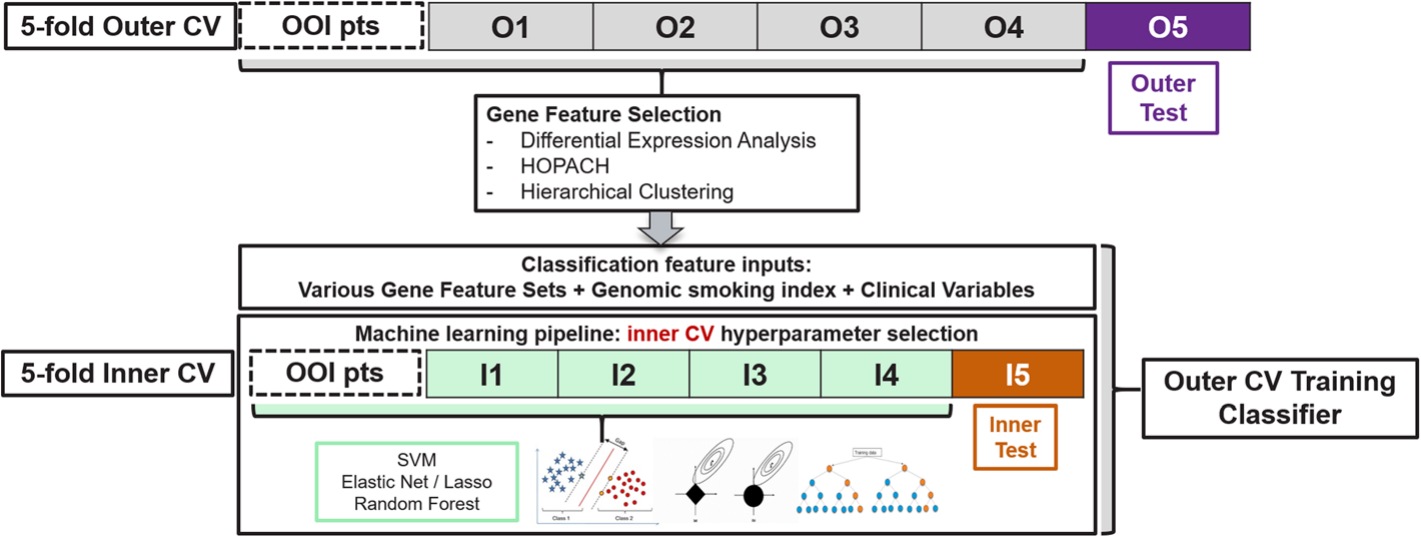
Analysis and evaluation pipeline based on a nested cross-validation schema

Within each CV loop, model training comprised three sequential steps (Fig. 1). (1) Feature selection by differential expression (DE) analysis for smoking status, which was performed using DESeq2 [14], with gender and cohort included in the design matrix. Genes differentially expressed by smoking status with FDR <  0.05 were selected into a feature set. (2) A feature reduction step to bring down the size of the feature set by a proportion of 0.1, 0.2 or 0.5 through HOPACH [23] or hierarchical clustering was also performed. Six additional feature sets were generated through this process. (3) At the model training step, hyperparameter optimization used an inner layer of 5-fold CV with five repeats. For each feature set, we examined two classification models: support vector machine (SVM) and logistic regression with elastic net. Ensemble models were also examined by combining individual machine learning methods via the weighted average of scores of individual models [24]. The final selected model was logistic regression using 248 gene predictors, trained on the entire training set (*N* = 1578) with elastic net regularization (hyperparameters *α* = 0.129, *λ* = 0.131). The genomic smoking index is the continuous logit score by the final model and was used as a continuous feature in benign vs. malignant classifiers.

### Cell populations in the bronchial samples

A bronchial brush collects airway epithelial cells, consisting of ciliated, secretory and basal cells, with possible contamination from blood and inflammatory cells [25, 26]. To estimate the cell populations of our samples, we used the single sample GSEA algorithm [27] to derive cell type indexes for three types of airway epithelial cells (basal, ciliated and secretory) [28], blood cells [29] and immune cells [27] respectively. Each index is the gene set enrichment score for respective cell-type-specific signature genes (Additional file 2).

### Genomic classifier for specimen collection timing

Specimen collection timing is a binary variable that describes whether the bronchial brushing sample is collected prior to or after other cytology and/or pathology sampling during bronchoscopy. We observed a correlation between specimen collection timing and cell type index. We used this insight to train a genomic classifier for specimen collection timing (prior vs. after), as a proportion of the training samples have this information missing and such a genomic classifier will provide the needed imputation. This classifier was developed using all BGC Registry training samples for which the collection timing variable was clinically recorded (*N* = 285). Input features were restricted to genes that were members of the airway epithelial, blood or immune cell signatures (Additional file 2). Classifier training utilized ten repeats of 5-fold cross-validation with four feature selection options as follows: no feature selection, differentially expressed genes with adjusted *p*-value < 0.05 with or without additional feature reduction by HOPACH or hierarchical clustering. Each feature set of the cross-validation training portion was fit into SVM, elastic net logistic regression and ensemble of all individual models with the same feature set (Fig. 1). The final classifier is a logistic regression model using 84 gene predictors. Its performance was validated on the test set reserved for the GSC benign/malignant lung cancer classifier, with a pre-defined decision boundary = 0.5 on the probability scale. Samples with higher probability score than the decision boundary are predicted as being collected “After”.

### GSC development

#### Genomic feature selection

Selection of gene expression features for GSC development involved multiple steps. As described above, an initial gene filtering step excluded low expressers and genes sensitive to batch effects. Second, the differential gene expression analysis step identified genes differentially expressed with respect to the benign/malignant label for lung cancer. Next, a feature reduction step was performed to trim down the gene feature set by a proportion of 0.1, 0.2 or 0.5 through HOPACH [23] or hierarchical clustering to remove highly correlated or redundant features. The final gene set combined with clinical covariates were provided as the input features for machine learning algorithms.

#### Differential expression analysis

For the genomic feature selection step described above, we identified genes differentially expressed between bronchial brushing samples from subjects with benign and malignant pulmonary nodules. We carried out differential gene expression analysis for cancer status (benign versus malignant) with DESeq2 [14]. Raw gene-level expression counts of 17,954 genes from the initial feature filtering step were used to perform the differential analysis. To control for variation associated with clinical factors, gender, clinical smoking status and cohort were included in the design matrix. To boost the sample size and the power of the DE analysis, we used an auxiliary set of out-of-indication malignant bronchial samples in addition to the within-indication training samples in the DE analysis. The auxiliary set was composed of bronchial samples from 579 AEGIS subjects and 41 subjects from an independent study [22]. These samples were out of indication due to lung malignancy diagnosed at the time of bronchoscopy and therefore included in the auxiliary set. The DE step of feature selection identified genes differentially expressed with respect to benign/malignant label with FDR <  0.05.

#### Imputation for missing values in clinical variables

Imputation was applied to generate values for clinical variables missing in the clinical records of some subjects, when those variables are under consideration as candidate classification features. These include years since quitting smoking, pack-years, nodule size and age. To impute those missing values, the median value was calculated for each cohort using subjects with non-missing values separately for within-indication and out-of-indication groups and further subdivided by physician assessed risk group (low, intermediate and high). Inhaled medication is a binary variable with “yes” indicating currently taking inhaled medication and “no” indicating not currently taking such medication; the default value was “no” if it was missing. For specimen collection timing, the genomic classifier for specimen collection timing was used to impute missing data.

#### Training the benign vs. malignant classifiers

For model training, the response variable was the adjudicated benign/malignant diagnosis label, and the predictive features comprised clinical features and normalized expression levels for differentially expressed genes. We examined three types of feature sets: clinical-dominant feature sets including age, genomic gender, pack years, specimen collection timing, inhaled medication use, and genomic smoking index; genomic-only feature sets including individual gene features and the genomic smoking index; and clinical-genomic feature sets that included clinical variables and gene features as well as interactions between the two. We evaluated multiple classification models, including random forest, support vector machine, linear discriminant analysis, gradient boosting and penalized logistic regression. We also examined ensemble models constructed as the weighted average of scores from individual classifiers.

Each classifier was evaluated using repeated 5-fold cross-validation. Sample partitions for cross-validation were balanced by adjudicated benign/malignant label, smoking status and specimen collection timing based on the prevalence in the overall training set. Hyperparameter tuning was performed within each cross-validation split in a nested manner [30] (Fig. 1). We used random search and one standard error rule for selecting the best hyperparameters from inner CV to minimize potential overfitting. Ultimately, hyper-parameter tuning was repeated on the entire training set to determine values to be used in the final classifier.

Our primary goal was to develop a high-sensitivity test that down-classifies low and intermediate pre-test risk patients to very-low and low post-test risk, respectively. For this purpose, we assessed classifier specificity when the decision boundary was set to achieve a minimum sensitivity of 0.9 on the sample set of within-indication subjects with low and intermediate pre-test risk. Individual and ensemble models were evaluated separately based on 5-fold CV with ten repeats. For each repeat of 5-fold CV, the cross-validated scores of within-indication samples with low/intermediate pre-test risk from the five folds are combined to evaluate the best specificity at minimum sensitivity of 0.9. Models were compared by the median and interquartile range (IQR) of specificities from the CV repeats.

Our secondary goal was to up-classify intermediate and high pre-test risk patients to high and very-high post-test risk groups, respectively. We evaluated the performance to the secondary endpoint with the maximum up-classification rate at the post-test positive predictive value (PPV) threshold of 0.65 for intermediate pre-test risk samples and PPV threshold of 0.9 for high pre-test risk samples. Post-test PPV was calculated using specificity, sensitivity and cancer prevalence in each pre-test risk group.

**Fig. 2:**
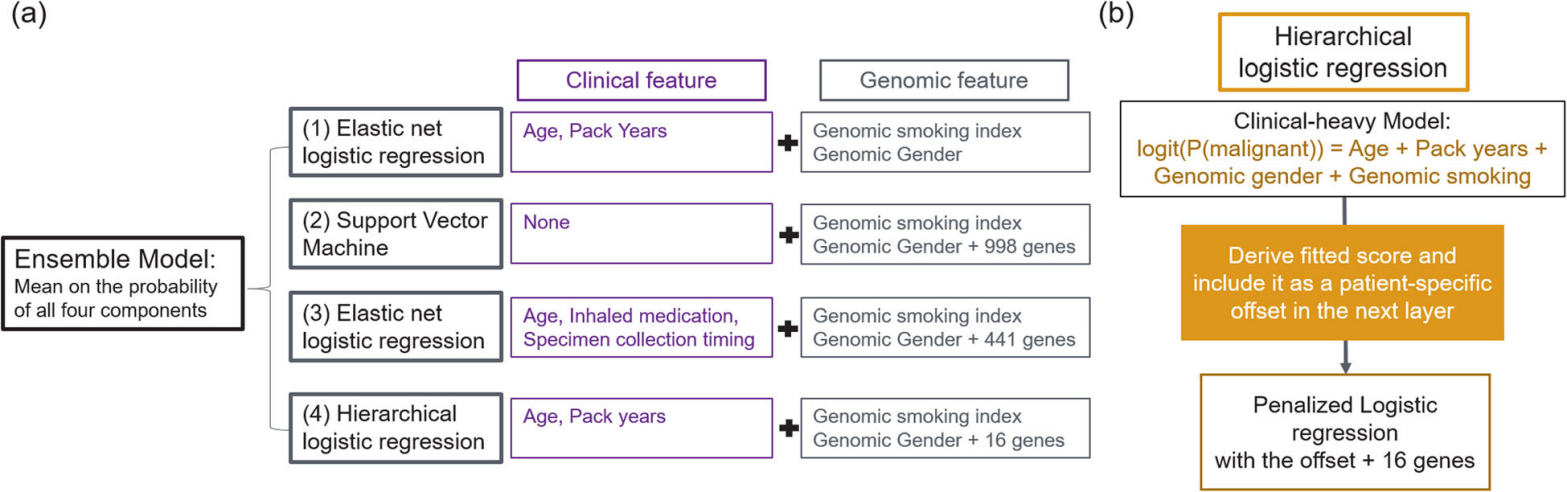
Genomic sequencing classifier structure. **a** Overall structure of the Ensemble model. **b** Detailed structure of the hierarchical logistic regression component

The final GSC is an ensemble model comprised of four individual models (Fig. 2). The first model is a clinical-dominant model, which is a penalized logistic regression with main effects and interaction effects from four clinical variables – age, gender, pack-years and genomic smoking index. The second model is a genomic-dominant linear-kernel SVM model, using gender, genomic smoking index and 998 genes as features. The third model is a clinical-genomic model, which is a penalized logistic regression with main effects and interaction effects from five clinical variables and 441 genes. Clinical variables in this model include age, genomic gender, inhaled medication, specimen collection timing and genomic smoking index. The fourth model is a hierarchical generalized linear model (GLM). The motivation for this approach is to estimate the association of gene expression with disease status after accounting for established clinical risk factors for lung cancer. At the top level, a clinical-dominant logistic regression model using main effects of clinical features was fit to subject labels. The logit score of this model was then used as an offset in the fitting of a second clinical-genomic model with main effects of clinical features and gene features using penalized logistic regression. We hypothesized that most gene expression changes associated with a clinical risk factor do not have a direct association with the development of cancer. By accounting for the cancer risk with clinical factors as much as possible at the top layer (a clinical-dominant model), we selected genes that have additional predictive power for lung cancer risk which are unlikely to be confounding with clinical factors in the second layer. The final hierarchical GLM model contained age, gender, pack-year, genomic smoking index and 16 individual gene features. The final ensemble model score is the logit of mean probabilities from the four individual models. Together, this ensemble model uses five clinical features (age, gender, pack-year, inhaled medication and specimen collection timing) and 1232 gene features (including genes used in genomic smoking index).

### Evaluation of technical batch effects

Replicated control samples and sentinels across all plates in the training and validation sets were evaluated in the genomic space and scored with candidate classifiers to estimate plate-to-plate variability using a linear mixed effect model controlling for biological effects from individual samples. The candidates that are associated with smaller score variabilities were preferred.

To measure the robustness of our candidate classifier against different enrichment probe lots, a key library preparation reagent, 71 brushing samples were re-sequenced using two different, independently manufactured lots of enrichment probes. These samples were selected from the training set to have scores that cover the candidate classifier score range. Each sample had three replicates, one in training and two additional runs with different enrichment probe lots. All replicates were scored with candidate classifiers to estimate score variability. For each new lot, a linear model of the scores was fit to the scores from development batches using paired replicates to estimate two forms of systematic bias in scores, e.g. score shift and rotation, associated with each enrichment probe lot.

### Independent validation

The final candidate classifier was validated on an independent test set. Three decision boundaries were optimized on respective pre-test risk groups in the test set to achieve these aims: 1) 90% sensitivity on the down-classification of intermediate and low pre-test risk subjects, 2) 90% PPV on the up-classification of high pre-test risk subjects to very high post-test risk, and 3) 65% PPV on the up-classification of intermediate pre-test risk subjects to high post-test risk. Classifier predictions were compared to the adjudicated subject label of benign or malignant to compute sensitivity and specificity. Cancer prevalence in the low (5%), intermediate (28.2%) and high (73.6%) pre-test risk groups were derived from the test set, and were applied, together with sensitivity and specificity, for the computation of NPV and PPV in each pre-test risk group. A receiver-operator characteristic curve (ROC) and corresponding area under the curve (AUC) were also produced using the intermediate and low pre-test risk subjects combined to evaluate the performance of the down-classification feature.

## Results

### Study cohorts

A set of 1237 subjects from AEGIS consisting of 189 within-indication and 1048 out-of-indication subjects, and 265 subjects from the BGC Registry consisting of 122 within-indication and 143 out-of-indication subjects, were used to develop our genomic sequencing classifier (Table 1). The independent test set consisted of brushing samples from 412 within-indication subjects: 246 were from AEGIS, all of which were used in the original BGC validation, and 166 were from BGC Registry. Clinical characteristics of the within-indication subjects from the training and test sets are summarized in Table 2. The composition with respect to key demographic and clinical factors was prospectively balanced between training and test sets. The test set is enriched with infiltrate lung lesions (*p* <  0.001) and non-small-cell lung cancer (*p* = 0.025), compared to the training set.

**Table 1:** Training and test set composition. ^a^ The OOI Malignant set includes subjects out of indication due only to having a positive lung cancer diagnosis from bronchoscopy. ^b^ The OOI Other set includes subjects that are out of indication due to other reasons (never smokers, concurrent or prior cancer or metastatic to lung)

	Set	Cohort	Pre-Test Risk Group				N
			Low	Intermediate	High	Missing	
Training (*N* = 1502)	Primary (Within Indication)	AEGIS	25	50	78	36	189
		Registry	7	80	35	.	122
		Total					311
	OOI Malignant^a^	AEGIS	1	24	477	77	579
		Total					579
	OOI Other^b^	AEGIS	48	122	217	82	469
		Registry	22	85	33	3	143
		Total					612
Test (*N* = 412)	Primary (Within Indication)	AEGIS	58	82	106	.	246
		Registry	22	106	38	.	166
		Total					412

**Table 2:** Demographic and clinical characteristics of training and test sets focusing on within-indication subjects

	Training		Test		***P***-value
Characteristic	AEGIS (***N*** = 189)	Registry (***N*** = 122)	AEGIS (***N*** = 246)	Registry (***N*** = 166)	
**Sex**					0.36
Female	72	65	83	84	
Male	117	57	163	82	
**Median age (IQR)**	62 (54–70)	64 (57–71)	62 (54–70)	65 (58–71)	0.45
**Race**					0.59
White	141	106	192	132	
Black	34	14	42	29	
Other	11	2	12	4	
Unknown	3	0	0	1	
**Smoking status**					0.45
Current	79	48	107	73	
Former	110	74	139	93	
**Median cumulative tobacco use (IQR)** – pack-year	40 (18–57)	35 (20–50)	35 (20–56)	35 (20–56)	0.82
**Lesion size**					**< 0.001**
Infiltrate	0	0	25	0	
< 2 cm	42	61	88	80	
2 to 3 cm	30	29	48	29	
> 3 cm	41	26	75	44	
Unknown	60	6	10	13	
**Lesion location**					0.47
Central	50	9	72	10	
Peripheral	78	107	108	144	
Central and peripheral	46	0	53	0	
Unknown	15	6	13	12	
**Lung-cancer histologic type**					**0.025**
Small-cell	8	3	8	1	
Non-small-cell	69	48	100	43	0.18
Adenocarcinoma	30	25	58	25	
Squamous	28	12	26	10	
Large-cell	6	1	4	0	
Non-small-cell not otherwise specified	5	10	12	8	
Other	0	2	0	2	
Unknown	21	3	3	6	
**Diagnosis of a benign condition**					**< 0.001**
Fibrosis	1	0	1	0	
Granuloma	15	6	26	10	
Infection	30	15	36	15	
Inflammation	4	2	1	2	
Multiple	6	0	8	0	
Other	17	4	25	2	
Resolution of Stability	18	39	38	40	
Clinically benign	0	0	0	45	

### Genomic feature correlation analysis

**Fig. 3:**
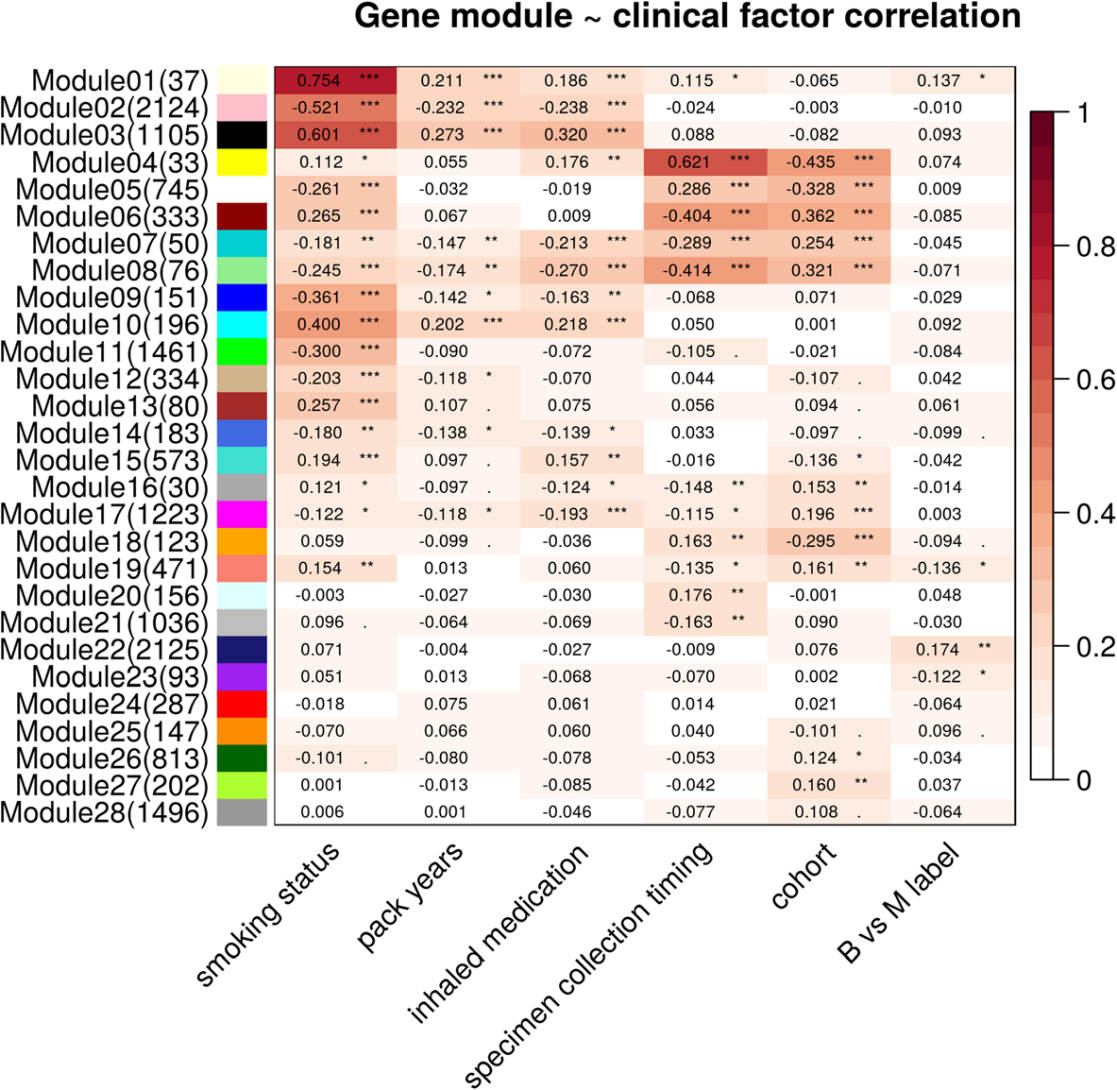
Gene correlation analysis (WGCNA): module eigengenes (listed by row) correlation with clinical factors (by column). Heatmap color is based on absolute Pearson correlation. Legend for *p*-value significance: '***' 0 <* p*-value ≤ 0.001; '**' 0.001 < *p*-value ≤ 0.01; '*' 0.01 < *p*-value ≤ 0.05; '.' 0.05 < *p*-value ≤ 0.1; ' ' 0.1 < *p*-value ≤ 1. Number of genes in each module is shown in parenthesis in row labels

**Fig. 4:**
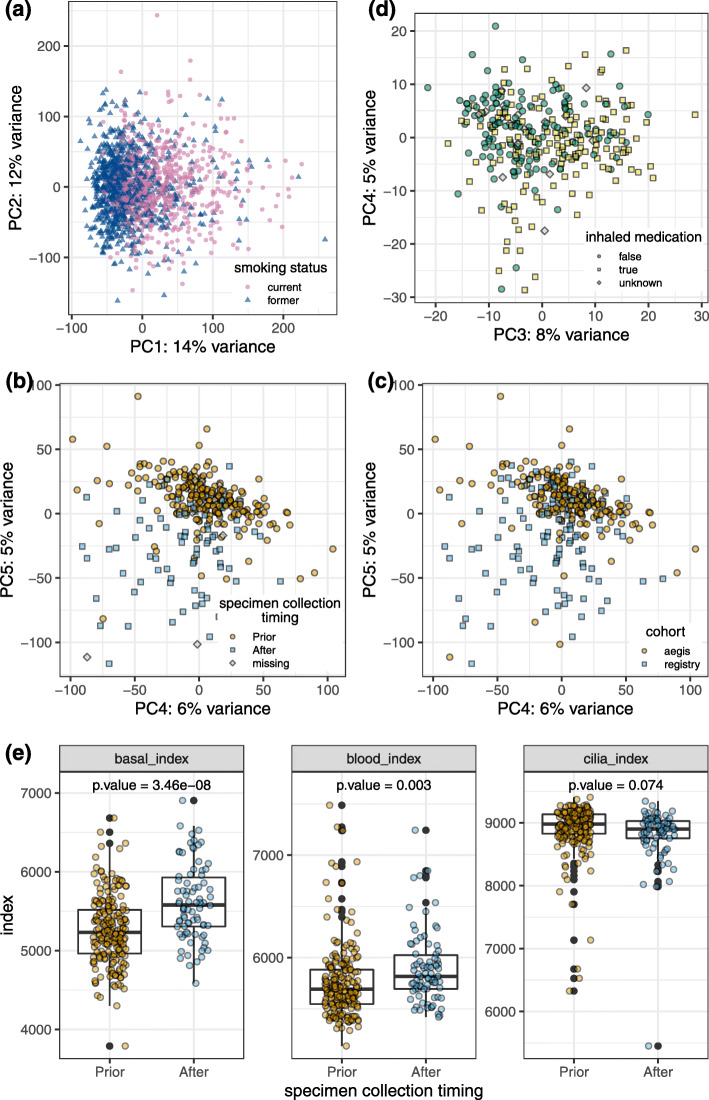
Gene expression variation associated with smoking status, specimen collection timing, cohort and inhaled medication. PCA was performed using scaled normalized (VST) expression data for **a** 17,954 genes from all subjects in the smoking index training set (*N* = 1578); **b** and **c** 17,954 genes from within-indication subjects in the training set (*N* = 311); **d** 998 benign vs malignant DE genes from within-indication subjects in the training set (*N* = 311). **e** Distribution of basal, blood, cilia and immune cell type indexes in within-indication subjects in the training set, separate by specimen collection timing

A total of 15,683 autosomal genes are clustered into 28 co-expression modules using the WGCNA gene clustering approach [19]. See Additional file 3 for overrepresented Gene Ontology biological processes associated with individual gene modules. We examined the correlation between module eigengenes (the first principal component of individual gene modules) and clinical factors of interest (Fig. 3). For 10 out of 28 co-expression modules, smoking status has a stronger correlation than all other clinical factors with the module eigengene, reflecting a strong genome-wide impact from cigarette smoking on gene expression. The impact of smoking status is observable in the first principal component of the gene expression profiles (Fig. 4a). Among these 10 modules, four modules also have significant correlation with smoking intensity as measured by pack-years. Specimen collection timing is the second strongest factor, associated with five modules, indicating an impact from the sample collection procedure on bronchial epithelial cell gene expression (Fig. 4b). Cohort is also a strong factor associated with three modules, reflecting the genomic differences between the two sample populations (Fig. 4c). Note that cohort and specimen collection timing are confounded, as 97% of AEGIS samples were collected prior to other cytology/pathology sampling while only 38% of BGC Registry samples were collected prior (Chi-square test p = 1.8 × 10^−27^). Eigengenes of 12 modules are significantly correlated with inhaled medication. These modules are also significantly correlated with smoking status or specimen-collection timing, possibly reflecting a shared cellular response to airway stimulants or perturbation. Modest sample separation by inhaled medication was observed in PCA space (Fig. 4d). Compared with other clinical factors, fewer gene modules are associated with lung cancer status, and the correlations are weaker as well. Similar results were observed through DE analysis. We performed DE analysis with respect to B/M disease label and clinical factors including smoking status, specimen collection timing and inhaled medication and observed far fewer differentially expressed genes by disease label than by the examined clinical factors (Additional file 1 Fig. S2).

### Smoking index

Smoking status (former vs. current) is manifested in bronchial airway gene expression (Fig. 4a). Therefore, we were able to develop a genomic classifier for smoking status with high accuracy: cross-validation performance in the training set showed a high AUC (median 0.956) and the performance is retained in the test set with an AUC of 0.945 (Additional file 1 Fig. S3). The smoking index not only captured the genomic signatures for smoking status, but also reflected smoking history and intensity, with a Pearson correlation of 0.23 with pack-years and − 0.61 with years since quitting smoking (Additional file 1 Fig. S3).

### Impact of specimen collection timing on cell type composition

With cell type indexes, we discovered that the gene expression of brushing samples can be largely impacted by the specimen collection timing. Specifically, compared to samples collected prior to other cytology and/or pathology sampling, samples collected after other sampling tend to have higher basal and blood cell gene expression, and lower ciliated cell gene expression (Fig. 4e). As the proportion of samples collected at the beginning of (prior to) the bronchoscopy in AEGIS and the BGC Registry cohorts is 97 and 38% respectively, gene expression in samples are largely different by collection timing and cohort at the whole genome level; this phenomenon is clearly manifested in the PCA analysis (Fig. 4b, c). Therefore, specimen collection timing was included as a binary variable in the benign vs. malignant classifier to help address the heterogeneous sample composition of our data. To impute missing values for the specimen collection timing variable, we developed a genomic classifier which achieved sensitivity of 0.81 and specificity of 0.81 with an AUC of 0.88 on 357 samples with truth labels for specimen collection timing in the independent test set.

### Cross-validation performance on the training set

We evaluated multiple methods of feature selection and machine learning algorithms on the primary within-indication set of 311 subjects in training. Overall, feature selection by DE analysis using the auxiliary sample set, composed of out-of-indication subjects such as “bronchoscopy positives”, generated models with better performance than models trained using primary within-indication subjects only. Clinical-genomic models outperformed clinical-only and genomic-only models. The addition of clinical-genomic interaction terms improved model performance over models with main effects alone. Linear models such as penalized regression model outperformed non-linear tree-based models such as random forest.

**Fig. 5:**
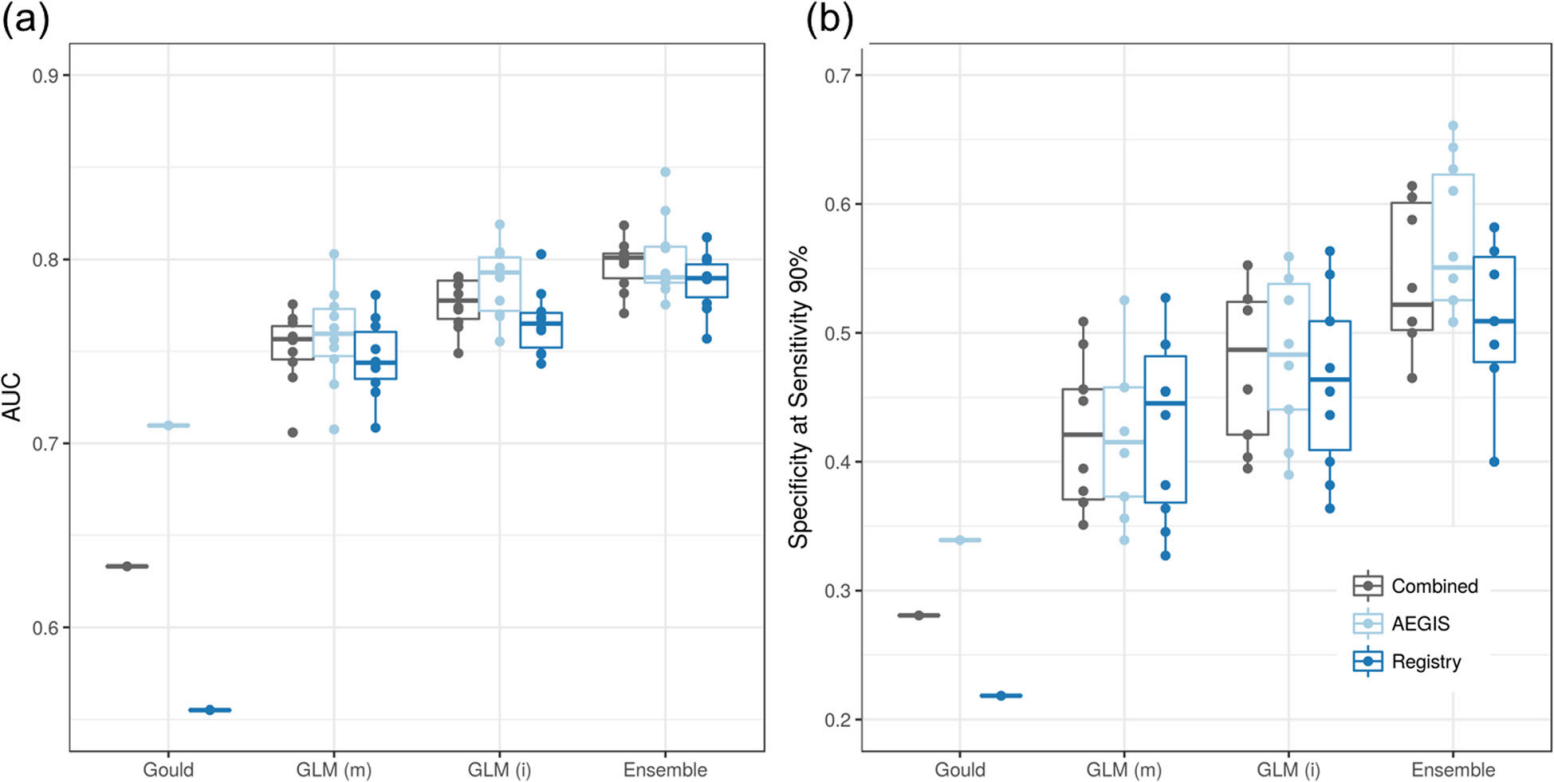
Cross-validation performance for down-classification. The performance is evaluated on within-indication training samples with low/intermediate pre-test risk (*N* = 162) using 10 repeats of 5-fold CV. The original Gould model was used to score training samples. GLM(m) is a generalized linear regression model only containing main effects of clinical features and genomic features. GLM(i) includes main effects and interactions between clinical features and genomic features. GLM(m) and GLM(i) used the same set of input clinical features: age, gender, nodule size, pack-year, years since quitting smoking, specimen collection timing and genomic smoking index. “Ensemble” is the final GSC classifier

The CV performance of four representative models for down-classification on low and intermediate pre-test risk samples (*N* = 162) in the training set is shown in Fig. 5. We used the Gould clinical model [31] as a comparator to estimate the probability of lung cancer in subjects with solitary pulmonary nodules using four clinical predictors: smoking history (pack-year), age, nodule size and time since quitting smoking. The Gould model had an overall AUC of 0.63 (0.71 for AEGIS and 0.55 for BGC Registry) and a specificity of 0.28 (0.34 for AEGIS and 0.22 for BGC Registry) at overall sensitivity of 0.9 (Fig. 5a and b). The low performance of the Gould model in our study may be due to the higher prevalence, heavier smoking history and smaller nodules in the VA Hospital population used to develop the Gould model, compared to the cohorts in this study [31].

A generalized linear model using only main effects of genomic features and clinical covariates (age, gender, nodule size, pack-year, years since quitting smoking, specimen collection timing and genomic smoking index) has a median CV AUC of 0.76 (0.76 for AEGIS and 0.74 for BGC Registry) and a specificity of 0.42 (0.42 for AEGIS and 0.45 for BGC Registry). (Fig. 5a and b, “GLM(m)). This model has an improved performance over the Gould model, and shows less performance difference between cohorts, indicating that genomic features may reduce the impact of demographic differences between cohorts on classification performance. Including interaction terms between clinical covariates and genomic features as predictors in the generalized linear model further improved performance, with a median CV AUC of 0.78 (0.79 for AEGIS and 0.77 for BGC Registry) and a specificity of 0.48 (0.48 for AEGIS and 0.46 for BGC Registry) (Fig. 5a and b, “GLM(i)).

We evaluated classifier performance in subgroups defined by individual clinical covariates. We observed a classifier score shift among subjects currently using inhaled medication; the shift is directionally consistent in both benign and malignant samples (Additional file 1 Fig. S4). The GLM model with clinical-genomic interactions showed a median score difference for inhaled medication that was 9–17% of the overall CV score range after controlling for benign/malignant label. Sensitivity (0.96 vs 0.88) and specificity (0.38 vs 0.65) also differed between subjects currently using and those not using inhaled medication. We therefore added a binary clinical covariate (subject currently using inhaled medication: Y/N) into the model. As a result, the median CV score difference by inhaled mediation was reduced to 4–5% of the overall CV score range, and sensitivity (0.93 vs 0.90) and specificity (0.47 vs 0.59) are stabilized. The overall down-classification performance also improved after adding inhaled medication into the classifier, with a median cross-validated AUC of 0.79 and specificity of 0.51 (Additional file 1 Fig. S4).

The final ensemble model achieved the best cross-validation (5-fold, 10 repeats) performance in the training set for the down-classification of low and intermediate pre-test risk subjects to very low and low post-test risk groups, with median cross-validated AUC = 0.80 (IQR = 0.017) and median specificity 0.52 (IQR 0.092) at sensitivity 0.92 (IQR = 0) on primary low/intermediate-risk samples (Fig. 5, “Ensemble”).

### Robustness of classifiers against technical assay variability

Within the same reagent lot, the total score variability of the final classifier estimated using repeated samples, sentinels and controls, is 0.123, which is 3.3% of the cross-validated score range (1%-trimmed range). The batch-to-batch variability is estimated as 0.058, which is a minor effect on the score variability.

Using 71 bronchial brushing samples repeated over three batches each with a different reagent lot, the estimated total score variability with different reagent lots is 0.16 (5.1% of the score range), with 0.068 contributed from reagent effects. Score variability in the final classifier is the lowest among candidate models, suggesting robustness to technical batch effects.

### Independent validation performance

In prospective validation, the final ensemble classifier achieved an AUC of 0.74 (95% CI: 0.67–0.81) on the combined low and intermediate pre-test risk groups with a sensitivity of 0.91 (95% CI: 0.81–0.97) and specificity of 0.45 (95% CI: 0.38–0.53) for down classification. Performance by pre-test risk group is listed in Table 3. This resulted in down-classifying 30% of subjects from pre-test intermediate to post-test low risk with post-test NPV of 91%; and 55% of subjects from pre-test low to post-test very low risk with post-test NPV of > 99%. Performance in individual cohorts are reported in Additional file 1 Table S1.

**Table 3:** Lung cancer genomic sequencing classifier validation performance (Down, up classification). ^*^Cancer prevalence calculation includes local benign subjects as Prevalence = $\frac{\# Malignant}{\# Malignant+\# Benign+\# Local\ Benign}$. The local benign subjects had local label as benign but did not have an adjudicated label. NPV, PPV and % Reclassified are all functions of prevalence (estimated including local benign subjects), sensitivity and specificity (both estimated excluding local benign subjects)

AUC	Pre-test Cancer Risk	^*^Cancer prevalence	Cancer risk re-stratification	Specificity	Sensitivity	Post-test NPV/PPV	^§^% Re-stratified
73.4% [68.3–78.4]	Low	5%	Low to Very Low	57.4% [44.8–69.3]	100% [39.8–100]	100% NPV [91.0–100]	54.5%
	Intermediate	28.2%	Intermediate to Low	37.3% [27.9–47.4]	90.6% [79.3–96.9]	91.0% NPV [80.8–96.0]	29.4%
			Intermediate to High	94.1% [87.6–97.8]	28.3% [16.8–42.3]	65.4% PPV [43.8–82.1]	12.2%
	High	73.6%	High to Very High	91.2% [76.3–98.1]	34.0% [25.0–43.8]	91.5% PPV [77.9–97.0]	27.3%

^§^% Reclassified (Low to Very Low, Intermediate to Low) = (1- Prevalence) specificity + Prevalence (1-sensitivity)

^§^% Reclassified (Intermediate to High, High to Very High) = Prevalence sensitivity + (1-Prevalence) (1- specificity)

^*^ There are 8, 33 and 4 local benign subjects in low, intermediate and high-risk group

For the secondary goal of up-classification, using additional decision boundaries based on pre-test risk category, the classifier up classified 12% of the intermediate pre-test risk subjects to high post-test risk with a positive predictive value of 65% (95%CI 44–82%). 27% of high pre-test risk subjects were reclassified to very high post-test risk with a positive predictive value of 92% (95% CI 78–97%). Detailed performance including sensitivity and specificity is listed in Table 3.

## Discussion

We describe the development and evaluation of the GSC, a second-generation algorithm for lung cancer risk stratification among patients with nondiagnostic bronchoscopy results. The GSC has expanded risk stratification utility compared to the first generation BGC, with accurate down- and up-classification of lung cancer risk in the intermediate pre-test risk group across several independent patient cohorts. The GSC down-classifies low and intermediate pre-test risk patients with a high negative predictive value (> 99 and 91% respectively) minimizing the need for additional invasive diagnostic procedures in patients reclassified to low risk. Up-classification of intermediate and high pre-test risk patients at high positive predictive value (65 and 91% respectively), can help with physician confidence, accelerating appropriate treatment and shortening time to diagnosis of cancer.

Challenges emerged during the development of GSC. We included multiple independent cohorts to increase sample size as well as to represent the heterogeneity in the contemporary clinical setting, including any changes in the use or diagnostic yield of lung nodule bronchoscopy over time. Several key clinical factors showed different distributions across the cohorts, some of which (smoking, age) appear to have an impact in the genomic space. To address this, we developed a genomic smoking index and proactively included it in the final classifier. The genomic smoking index not only captures the difference between current and former smokers, but also captures differences in smoking history, showing a positive correlation with pack-years and a negative correlation with years since quitting smoking. Different sample collection practices also created challenges. Sample collection prior to or after other bronchoscopic sampling has a substantial impact on the overall gene expression profile in the collected airway epithelial sample. One possibility is that prolonged external disturbance of the bronchial airway during bronchoscopy may cause physical damage to the bronchial epithelium, especially the ciliated cells lining the surface of airway tracts, and lead to bleeding. In line with this hypothesis, we observed more expression of blood cell signature genes and less expression of ciliated cell signature genes in samples collected at the end of bronchoscopy. To account for this impact, we included specimen collection timing as a feature during the machine learning algorithm development and observed improved performance.

In comparison to interfering factors, such as the clinical and technical characteristics of each cohort, the use of inhaled medication and specimen collection timing, the expression signals associated with lung cancer status (Y/N) are much weaker. We approached the challenge of distilling signals associated with lung cancer status while controlling for interfering factors by using novel strategies for feature selection and model construction. We created clinical-dominant feature sets, genomic-only feature sets and genomic-dominant feature sets. In clinical-dominant feature sets, we incorporated interactions among clinical covariates and the genomic smoking index, which helped resolve the confounding relationship between features. In genomic-dominant feature sets, we adjusted for interfering clinical factors in two ways. The first was to incorporate interactions between clinical covariates and gene expression levels, the other was to use a hierarchical structure where the genomic component was fit downstream of the clinical component. Incorporating genomic and clinical interaction terms improved training performance over methods only considering main effects. Multiple machine learning algorithms were examined on each feature set, and the best performing models on individual types of feature sets were then combined into an ensemble model. Machine learning algorithms built on different types of feature sets thus extract information on disease status from different angles. This strategy showed consistent performance across technical replicates and most importantly, retained predictive accuracy in an independent validation set consisting of different cohorts.

There are several important limitations to this study. First, the sample size in training and validation sets are limited. Although the sample sizes of both training and validation sets have increased substantially over previous studies [4, 7], sample size in certain subgroups remain small, limiting our ability to best capture signals in those subjects. Second, we optimized the decision boundaries and reported the performance on each pre-test risk group of the test set, also separately for up- or down-classification. This is mainly due to the limitation of sample size after filtering subjects with missing data and dividing the test set by pre-test risk groups. Third, our understanding of how certain factors impact the genomic signal is still limited. These include clinical risk factors for lung cancer such as age, gender and smoking history, as well as interfering factors such as inhaled medication and specimen collection timing. Additional clinical risk factors for lung cancer exist but are not captured in this study, such as environmental exposure and dietary habits [32]. The final model with only four clinical variables was chosen under these two considerations 1) the difficulty of collecting high-quality clinical data in real world setting and 2) similar performance between the final model with only four clinical variables versus more complex models including more clinical variables, Future studies may shed light on how these factors cause gene expression changes in bronchial airway epithelial cells. With a deeper understanding of underlying mechanisms, we may better delineate gene expression signatures specifically associated with lung cancer from these confounding signals.

## Conclusions

We developed a robust Genomic Sequencing Classifier (GSC) for lung cancer risk stratification with consistent performance across multiple independent test cohorts. The GSC shows durable performance in the face of demographic composition shift in clinical cohorts, and indirect and weak cancer signal compared with interfering technical factors. The GSC provides actionable information through down-classifying and up-classifying patient lung cancer risk with high NPV and PPV respectively. Among low and intermediate risk patients with down-classified risk, additional invasive diagnostic procedures could potentially be avoided; among intermediate and high-risk patients with up-classified cancer risk, the diagnostic process may be accelerated to inform next steps and reach definitive diagnoses sooner.

## Supplementary information

**Additional file 1 Fig. S1**: Consort diagram of training and validation sets. **Fig. S2**: DE analysis on B vs M, smoking status, specimen collection timing, and cohort. **Fig. S3**: Smoking index score distribution on test set. (a) Box plots of smoking index for smoking status. Scatter plots of smoking index distribution vs smoking-related variables, (b) pack-years and (c) years since quitting smoking. The smoking index is positively correlated with pack-years (Pearson’s correlation = 0.23) and negatively correlated with years since quitting smoking (Pearson’s correlation = − 0.61). **Fig. S4**: Cross validated model score distribution and model performance by inhaled medication. Model GLM(i + I) is similar to GLM(i), except that the additional clinical feature -- subject currently taking inhaled medication -- is included in the input feature set as main effect and for interactions with genomic features. (a) CV score distribution for within-indication low/intermediate pre-test risk samples, shown separately by subject label and inhaled medication. (b) CV performance for down-classification of within-indication low/intermediate pre-test risk samples. **Fig. S5**: Pairwise scatter plot of individual model in the final ensemble classifier for samples in test set. Benign and malignant samples are colored in blue and red respectively. The four models are (1) clinical heavy logistic regression model (2) genomic-only SVM model (3) clinical-genomic logistic regression model and (4) hierarchical logistic regression model.

**Additional file 2.** Cell type signature gene lists.

**Additional file 3.** Gene Ontology biological processes overrepresented in gene modules identified from the gene co-expression network.)

## Data Availability

All data generated and supportive for the conclusion in this study are included in this published article.

## Abbreviations

BGC: Bronchial Genomic Classifier
AEGIS: Airway Epithelial Gene Expression in the Diagnosis of Lung Cancer
GSC: Genomic sequencing classifier
CI: Confidence interval
CT: Computed tomography
OOI: Out of indication
VST: Variance stabilizing transformation
WGCNA: Weighted Gene Co-expression Network Analysis
CV: Cross validation
DE: Differential expression
SVM: Support vector machine
IQR: Interquartile range
PPV: Positive predicative value
NPV: Negative predicative value
GLM: Generalized linear model
AUC: Area under the curve
PCA: Principal component analysis
