# Supplementary material for: Improving lung cancer risk stratification leveraging whole transcriptome RNA sequencing and machine learning across multiple cohorts
Source: BMC Med Genomics. 2020 Oct 22;13(Suppl 10):151. doi: 10.1186/s12920-020-00782-1 (PMC7579926; doi:10.1186/s12920-020-00782-1)
Supplement: Supplementary file 1 — Additional file 1 Fig. S1: Consort diagram of training and validation sets. Fig. S2: DE analysis on B vs M, smoking status, specimen collection timing, and cohort. Fig. S3: Smoking index score distribution on test set. (a) Box plots of smoking index for smoking status. Scatter plots of smoking index distribution vs smoking-related variables, (b) pack-years and (c) years since quitting smoking. The smoking index is positively correlated with pack-years (Pearson’s correlation = 0.23) and negatively correlated with years since quitting smoking (Pearson’s correlation = − 0.61). Fig. S4: Cross validated model score distribution and model performance by inhaled medication. Model GLM(i + I) is similar to GLM(i), except that the additional clinical feature -- subject currently taking inhaled medication -- is included in the input feature set as main effect and for interactions with genomic features. (a) CV score distribution for within-indication low/intermediate pre-test risk samples, shown separately by subject label and inhaled medication. (b) CV performance for down-classification of within-indication low/intermediate pre-test risk samples. Fig. S5: Pairwise scatter plot of individual model in the final ensemble classifier for samples in test set. Benign and malignant samples are colored in blue and red respectively. The four models are (1) clinical heavy logistic regression model (2) genomic-only SVM model (3) clinical-genomic logistic regression model and (4) hierarchical logistic regression model. [file 12920_2020_782_MOESM1_ESM.docx]

**Supplementary Document**

**Figure S1**: Consort diagram of training and validation sets


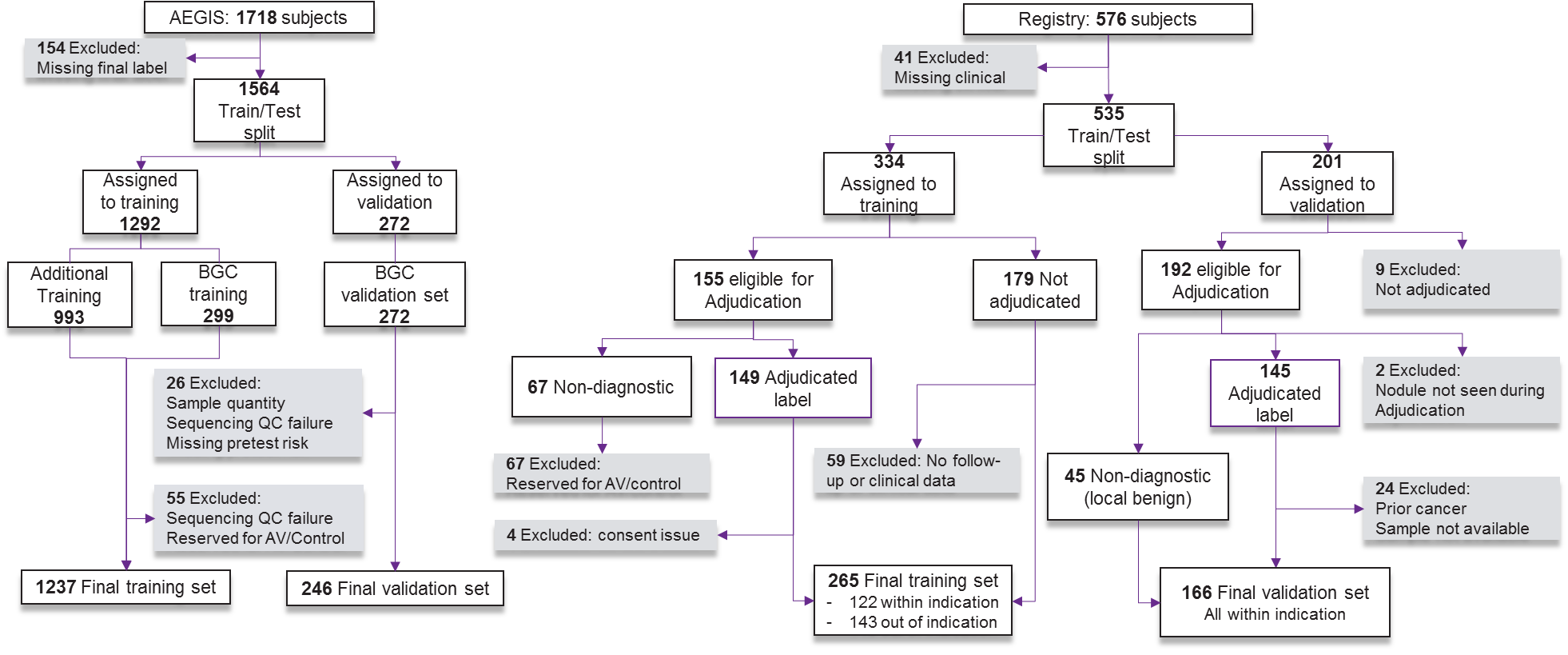


**Figure S2**: DE analysis on B vs M, smoking status, specimen collection timing, and cohort.


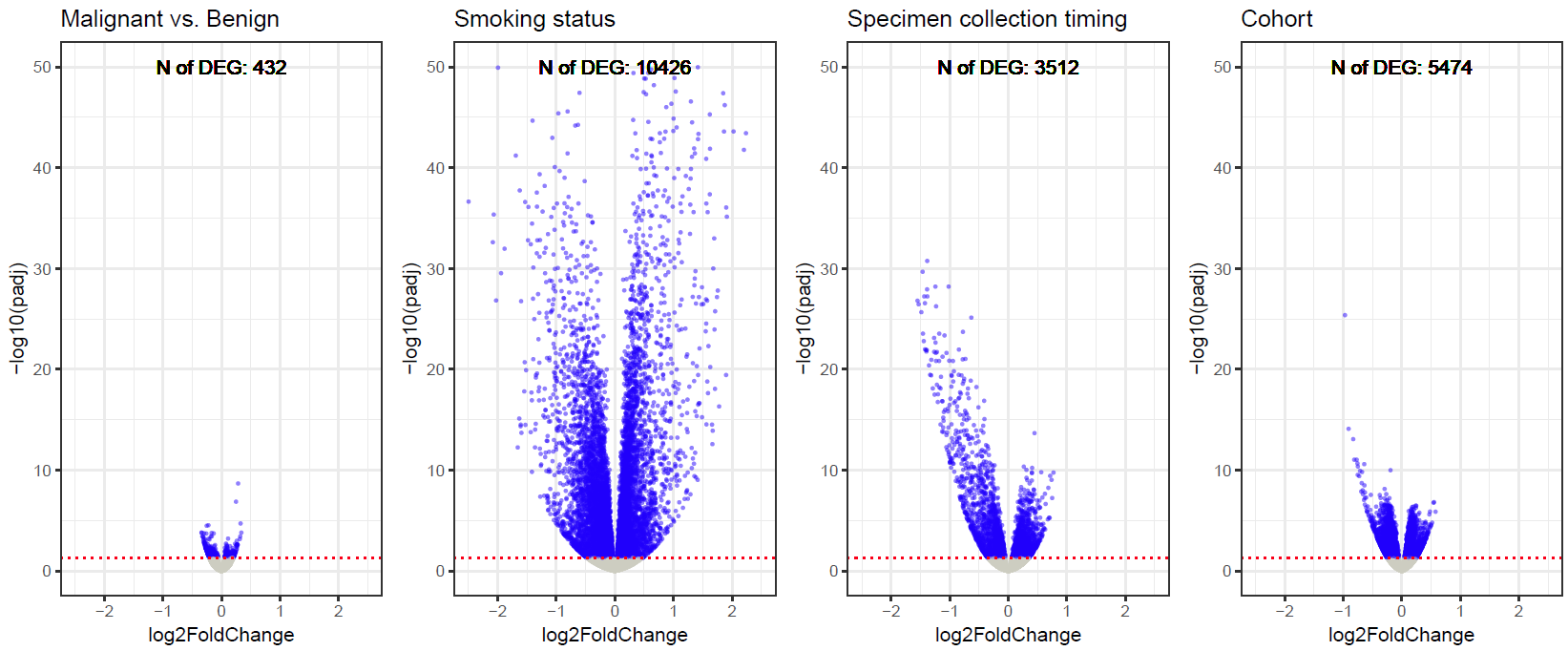


**Figure S3**: Smoking index score distribution on test set. (a) Box plots of smoking index for smoking status. Scatter plots of smoking index distribution vs smoking-related variables, (b) pack-years and (c) years since quitting smoking. The smoking index is positively correlated with pack-years (Pearson’s correlation = 0.23) and negatively correlated with years since quitting smoking (Pearson’s correlation = -0.61).


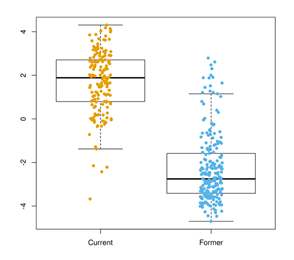

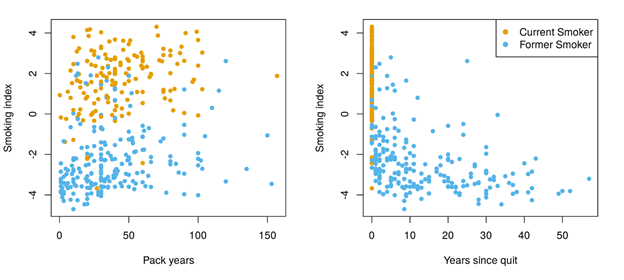
(a) (b) (c)

**Figure S4**: Cross validated model score distribution and model performance by inhaled medication. Model GLM(i+I) is similar to GLM(i), except that the additional clinical feature -- subject currently taking inhaled medication -- is included in the input feature set as main effect and for interactions with genomic features. (a) CV score distribution for within-indication low/intermediate pre-test risk samples, shown separately by subject label and inhaled medication. (b) CV performance for down-classification of within-indication low/intermediate pre-test risk samples.


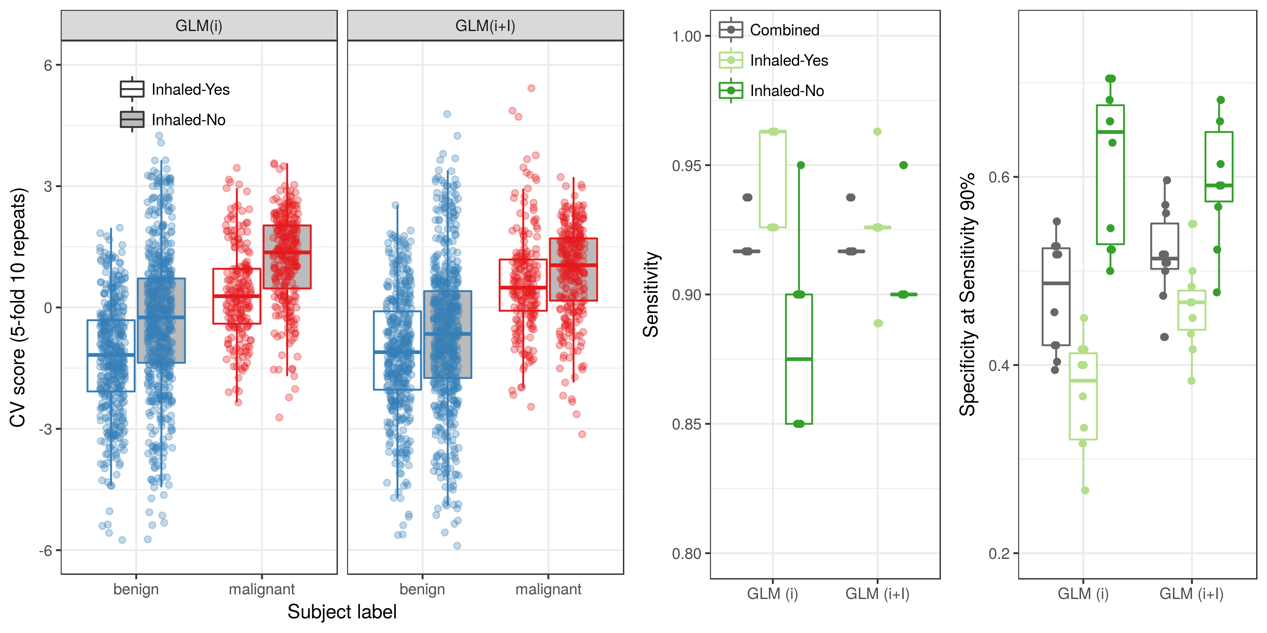
 (a) (b)

**Figure S5**: Pairwise scatter plot of individual model in the final ensemble classifier for samples in test set. Benign and malignant samples are colored in blue and red respectively. The four models are (1) clinical heavy logistic regression model (2) genomic-only SVM model (3) clinical-genomic logistic regression model and (4) hierarchical logistic regression model.

**
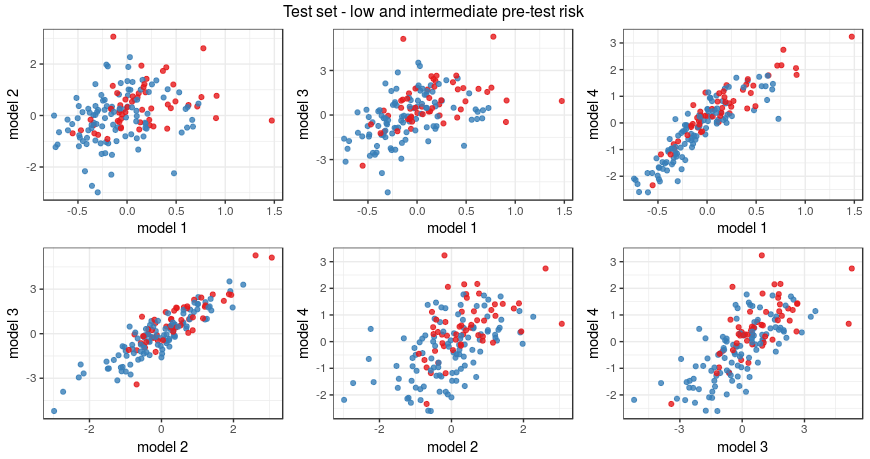
**

**
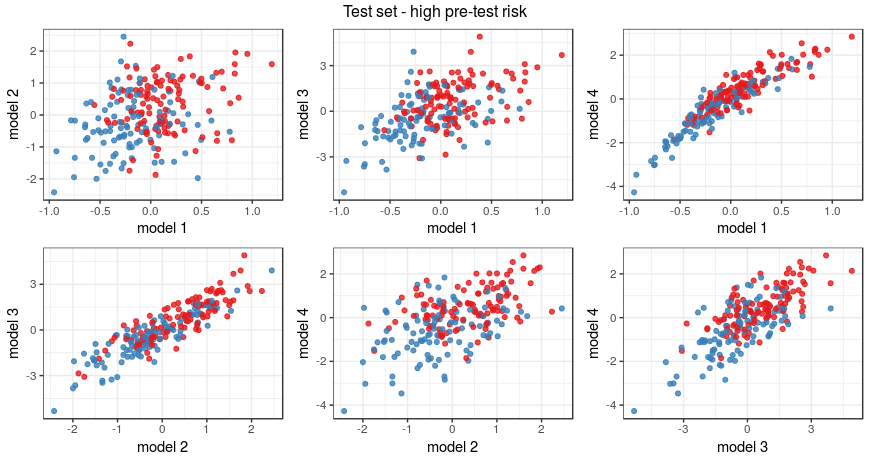
**

**Table S1.** Lung cancer genomic sequencing classifier validation performance by cohort (down, up classification). *Calculation of Cancer prevalence, NPV, PPV and % Re-stratified are as described in Table 3.

| Cohort | AUC | Pre-test Cancer Risk | *Cancer prevalence | Cancer risk re-stratification | Specificity | Sensitivity | Post-test *NPV/PPV | *% Re-stratified |
| --- | --- | --- | --- | --- | --- | --- | --- | --- |
| AEGIS | 73.7%  [67.6 – 79.8] | Low | 3.4% | Low to  Very Low | 55.4%  [41.5 – 68.7] | 100%  [15.8 – 100] | 100% NPV  [88.8 – 100] | 53.4% |
|  |  | Intermediate | 29.3% | Intermediate to  Low | 34.5%  [22.5 – 48.1] | 91.7%  [73.0 – 99.0] | 90.9% NPV  [71.7 – 97.5] | 26.8% |
|  |  |  |  | Intermediate to  High | 94.8%  [85.6 – 98.9] | 33.3%  [15.6 – 55.3] | 72.7% PPV  [43.6 – 90.2] | 13.4% |
|  |  | High | 80.2% | High to  Very High | 90.50%  [69.6 – 98.8] | 34.1%  [24.2 – 45.2] | 93.5% PPV  [79.0 – 98.2] | 29.2% |
| Registry | 72.6%  [63.7 – 81.5] | Low | 9.1% | Low to  Very Low | 66.7%  [34.9 – 90.1] | 100%  [15.8 – 100] | 100% NPV  [63.1 – 100] | 60.6% |
|  |  | Intermediate | 27.4% | Intermediate to  Low | 40.9%  [26.3 – 56.8] | 89.7%  [72.6 – 97.8] | 91.3% NPV  [77.3 – 97.0] | 32.5% |
|  |  |  |  | Intermediate to  High | 93.2%  [81.3 – 98.6] | 24.1%  [10.3 – 43.5] | 57.1% PPV  [27.3 – 82.6] | 11.6% |
|  |  | High | 55.3% | High to  Very High | 92.3%  [64.0 – 99.8] | 33.3%  [14.6 – 57] | 84.3% PPV  [42.6 – 97.5] | 21.9% |
